# A Manual of Diseases of the Nervous System

**Published:** 1888-11

**Authors:** 


A Manual of Diseases of the Nervous System. By W. R. Gowers, M. D., F. R. C. P., Assistant Professor of Clinical Medicine in University College, London. * * American Edition. With three hundred and forty-one illustrations. Philadelphia, Pa. P. Blakiston, Son & Co. 1888.

The scope and purpose of this book are well expressed in a paragraph of the preface to the American edition: "The object of the following work is to afford the student the means of gaining an adequate conception of our present knowledge of the diseases of the nervous system, and to supply the practitioner with the information he needs for dealing with those diseases in his daily work. I have endeavored, therefore, to present to the reader the lessons taught by observation, rather than the details of the cases observed, to subordinate, as far as possible, theory to fact, the speculative to the practical, and to employ hypothesis only where it is indispensable for the proper grasp of facts."

The want of such a work is felt by every general practitioner of medicine. Diseases of the nervous system are more obscure, are less studied, and information in regard to them more difficult to procure than any other class of maladies to which his attention is directed. The physiology of nervous action is not perfectly understood; the pathology of its numerous diseases, whether of structure or function, no better; and the protean and erratic symptoms by which they are indicated often unintelligible. The familiar text-books on surgery or the practice of medicine are often silent, or contain but meagre reference to them. A book, therefore, within reasonable cost and limits, which furnishes to the reader a clear and intelligible account of them, together with the most rational methods and means of treating them, is a *desideratum*, and will be appreciatively welcomed by the medical public.

